# Quality of life among caregivers of sickle cell disease patients: a cross sectional study

**DOI:** 10.1186/s12955-018-1009-5

**Published:** 2018-09-10

**Authors:** Badr M. Madani, Rajaa Al Raddadi, Soad Al Jaouni, Mohab Omer, Marie-Isabelle Al Awa

**Affiliations:** University of Jeddah, Asfan road, Jeddah, 23881 Saudi Arabia

**Keywords:** Sickle-cell disease, Caregivers, Parents, Quality of life, Satisfaction; Saudi Arabia

## Abstract

**Purpose:**

To assess the quality of life (QoL) of caregivers of children with sickle cell disease (SCD) and to determine the risk factors associated with poor QoL.

**Method:**

A cross sectional study was conducted between 01 and 30 June 2015, in a tertiary care center in western Saudi Arabia to assess various dimensions of QoL by using TNO-AZL Questionnaire for Adult’s Health-related Quality of Life (TAAQOL). A total 164 adult caregivers (aged 16 years or more) of children with SCD, who were regularly visiting the department were enrolled (refusal rate = 61.6%). The questionnaire scores were transformed into 0–100 scale; with higher scores indicating less difficulty and better QoL Demographic, socioeconomic data and a satisfaction questionnaire regarding participants’ lifestyle were collected and analyzed as risk factors for impaired QoL, by comparing different QoL dimensions’ scores using independent *t*-test, Oneway ANOVA, or linear regression, as appropriate.

**Results:**

Sixty-three caregivers were included; 79.4% were mothers, age range 21–71 years, 64.5% were from low social class receiving insufficient support and financial needs were unmet for considerable number of families. Analysis of QoL using TAAQOL showed that emotions (median [75th centile] = 44.44 [66.67] for negative and 61.11 [72.22] for positive emotions), sleep quality (66.67 [91.67]) and sexual life (50.00 [83.33]) were the most affected dimensions. Professional achievement (91.67 [100]), cognitive skills (83.33 [100]), and social contact (100 [100]) were relatively preserved. Negative emotions were more marked in mothers and mostly predicted by satisfaction with social relations notably with partner (B = 3.14, *p* = 0.016), friends (B = 2.51, *p* = 0.015) and relatives (B = 2.69, *p* = 0.016). Positive emotions were predicted by the levels of satisfaction of the caregiver with his/her health (B = 2.56, *p* = 0.001), job achievement (B = 4.54, p = 0.001), living conditions (B = 2.60, *p* = 0.034) and the condition of the diseased child (B = 2.55, *p* = 0.011). A strong correlation was found between sleep quality and cognitive skills.

**Conclusion:**

There are notable financial and emotional burdens on the caregivers of children with SCD affecting various aspects of their QoL, which are likely to be impacted by the individual levels of social and professional achievement. Physicians and health authorities should give particular attention to the QoL of caregivers and families of children with SCD, to help them cope up with the disease and overcome its related psychological and financial impacts.

**Electronic supplementary material:**

The online version of this article (10.1186/s12955-018-1009-5) contains supplementary material, which is available to authorized users.

## Background

World Health Organization estimated that over 5% of the world population carries genes of hemoglobinopathies. Approximately 23 out of 10,000 people are affected with sickle cell disease (SCD), with the highest prevalence in African countries, 110 out of 10,000 people [1]. However, SCD is also relatively prevalent in some cultures with high rates of consanguinity and large family size, such as in the Middle-East region, including Saudi Arabia, where hemoglobinopathies constitute real public health issues [2, 3]. SCD is one of the most common genetic disorders in Saudi Arabia, with an overall prevalence rate of 44.1 (42 carriers and 2.1 cases) per 1000 and marked regional variations reaching up to 134.1 per 1000 in the Eastern Region [3, 4].

SCD is a hemoglobinopathy characterized by a chronic anemia with various acute “painful crises” and chronic symptoms that require daily care and sometimes intensive medication; all negatively impacting physical functioning, sleep, school performance, and overall quality of life (QoL) of patients [5, 6]. An increasing interest to the various aspects of patients’ QoL is noted in the recent publications, highlighting the contribution of different factors such as complications, comorbidities, compliance to treatments, etc. [7, 8].

On the other hand, taking care of a child with SCD is a challenging experience for both the caregiver and the other family members [1, 2, 9]. Since, the management of SCD should be family-centered [10], besides the total dependence of the afflicted child on his/her caregiver for general care and treatment, parents, or caregivers, are subjected to continuous pressure; which may, in return, affect the patient’s QoL [11]. Consequently, caregivers are developing psychological disorders such as depression and anxiety [2, 11–14]. Several studies have described the association between parenting a child with SCD and psychological distress; approximately 30 to 40% of caregivers had symptoms of psychological distress [15–17].

Generally, common genetic disorders including SCD, constitute a considerable emotional and financial burden on patients and families, and even on the society, particularly in developing countries [18, 19]. A study from Nigeria, where the disease is highly prevalent, demonstrated that caregivers were exposed to intense pressure with the risk of developing psychological problems; which affected their ability to take care the afflicted children. Caregivers were in crucial need for attention and all kinds of support by both the physicians and authorities [19]. Another Nigerian study exposed a triangular relationship between the patient, the disease and the family. Exclusive caregivers, usually mothers, were the most likely to suffer from psychological problems and physical distress [18].

In developed countries such as Netherlands, female caregivers of children with SCD had significant impairment in the QoL, compared to healthy females and female caregivers of healthy children. All dimensions of the QoL were affected, particularly mood, sleep quality, feeling of happiness, and cognitive functioning [11].

In Saudi Arabia, despite high prevalence of the disease, no comprehensive data about health-related QoL of parents and/or caregivers of patients with SCD has been published so far. Such data will provide an insight that is necessary for optimizing the management of patients with SCD, by opening complementary perspectives to the purely medical follow-up and offering more targeted and specific solutions.

This study was conducted to investigate the QoL of caregivers of children with SCD in Western Saudi Arabia, and to assess the true impact of the illnesses on their overall well-being. Different dimensions of the QoL of caregivers were explored as well as the risk factors associated with deteriorated QoL. The dimensions of QoL that are more affected by the disease and the sub-groups of caregivers who are most exposed to poor QoL outcomes, with regards to the risk factors, were defined. We hypothesized that worse child conditions and lower satisfaction regarding different life domains may be significantly impacting the caregivers’ QoL.

## Methods

### Population and setting

A descriptive and analytic cross-sectional study was conducted between 01 June 2015 and 30 June 2015, in King Abdul Aziz University Hospital (KAUH), Jeddah, Kingdom of Saudi Arabia. Parents of the 164 children (aged < 16 years) with SCD following at KAUH were identified using the hospital database and invited to present at the outpatient clinic of KAUH to participate in the study. In addition, adult caregivers of children with SCD visiting the outpatient clinic of KAUH during the study period were directly approached and solicited to respond to the study questionnaire, in case, they had not received the invitation. All eligible and consenting respondents from either method were included. Inclusion criteria were applied as follows: parents, uncles, aunts, brothers or sisters aged 16 years or over, caregivers of a pediatric patient (aged up to 16 years) with a SCD diagnosed > 1 year ago and registered for treatment in KAUH. There was no restriction on gender, nationality or race to participate in the study. Exclusion criteria were applied as follows: caregivers who did not understand Arabic or English.

### Study tool

Caregivers were interviewed using the TNO-AZL Questionnaire for Adult’s Health Related Quality of Life (TAAQOL questionnaire), a validated questionnaire developed by the Dutch Institute of Prevention and Health and the Leiden University Hospital (TNO-AZL) [20]. It is a systematic tool to measure health problems or limitations and their impact on general well-being. The questionnaire was translated from Dutch into Arabic, although English version was used for data processing. This questionnaire was chosen because it enables assessing various dimensions of the quality of life that may be separately or mutually impacted. A written permission from the principal author was obtained to use the questionnaire. The questionnaire is divided into 9 subscales, each assessing one of the following dimensions of the QoL: mobility, fine motor skills, cognitive skills, sleep quality, pain, social isolation, job achievement, sexual life, and emotions. Each of these subscales contains 2 to 15 items; each item is answered on a 0 to 3 difficulty scale: 3 = no difficulty; 2 = a little; 1 = some; 0 = a lot. Item scores are added within each subscale; and raw subscale scores are transformed into 0–100% scores; with higher scores indicating less difficulty and better QoL. Transformed scores were analyzed as continuous variables.

In addition, participants were investigated regarding the following: 1) demographic and socioeconomic data, 2) characteristics of the care giving (type, frequency), 3) any type of psychological or financial support received, 4) daily free time management, and 5) participants’ satisfaction regarding various aspects of life (e.g. health condition, job achievement, relationships, etc.).

### Data collection method

The questionnaire was distributed in the outpatient clinics, emergency department (ER), pediatric wards, and daycare department during the study period week days. A team of residents including the author took on the mission to administer the questionnaire to eligible participants. Given the extent of the interview (30 to 45 min to complete all questionnaire items), a break was allowed for the participants at any time they suggested.

### Ethical clearance

The study protocol was approved by the ethical committee of the Joint Program of Family and Community Medicine (JPFCM) and by the institutional review board of KAUH.

### Statistical methods

Statistical analysis was carried out using SPSS, version 21 (IBM SPSS Statistics for Windows, Armonk, NY: IBM Corp. 2012). Descriptive statistics were used to present the patterns of participant’s answers to the different items of the questionnaire; where continuous data were presented as mean ± standard deviation and categorical data were presented as frequency (percentages). Preliminary analysis showed that transformed scores of QoL dimensions showed are not normally distributed, using both Kolmogorov-Smirnov and Shapiro-Wilk tests. Consequently, to analyze risk factors for impaired QoL, correlations with demographic and socioeconomic parameters were analyzed using nonparametric tests including Mann-Whitney U test and Kruskal-Wallis tests, as appropriate. Results were presented as median and range. In case if the risk factors were numerical variables (e.g. age, number of children, lifestyle satisfaction scores), linear regression analysis was used. Linear regression was also carried out to analyze life domains satisfaction scores as predictors for positive and negative emotions. Results are presented as unstandardized regression coefficient (B) with significant level. A *p*-value < 0.05 was fixed to reject the null hypothesis.

## Results

### Demographic and socioeconomic data

Sixty-three caregivers agreed to participate among 164 regularly visiting the outpatient clinic of KAUH (participation rate = 38.4%). Of these 50 (79.4%) were mothers and 7 (11.1%) were fathers; mean ± SD (range) age was 39.5 ± 9.8 (21–71) years. Almost two-third (64.5%) of the families lived with a monthly income < 1282 USD and only 10 (16.7%) had registered insurance. Socio-demographic and economic data of the caregivers of children with SCD in Western region of Saudi Arabia is presented in Table 1.

**Table 1:** Socio-demographic and economic data of the caregivers of children with SCD in Western region of Saudi Arabia

Variables	Frequency	Percentage (%)
Family relationship with the patient		
*Father*	7	11.1
*Mother*	50	79.4
*Sister*	4	6.3
*Brother*	2	3.2
Marital status		
*Single*	6	9.5
*Married*	55	87.3
*Widowed*	2	3.2
Children age category		
*At least one child < 5 years old*	18	28.6
*All children aged > 5 (5–14) years*	45	71.4
Educational level (participant)		
*Illiterate*	13	20.6
*Intermediate or high school*	32	50.8
*University of higher*	18	28.6
Educational level (partner)		
*Illiterate*	5	8.9
*Intermediate or high school*	38	67.9
*University of higher*	13	23.2
Job condition		
*Employed*	15	23.8
*Looking for job*	1	1.6
*Student*	4	6.3
*Housewife*	40	63.5
*Free work*	1	1.6
Reason of no job		
*In relation with the patient’s care or health problems*	21	56.8
*Other reason*	9	24.3
Monthly family income (USD)^a^		
*less than 1330*	40	64.5
*1330-2660*	11	17.7
*2660-4000*	7	11.3
*more than 4000*	4	6.5
Income resources		
*Salary, company dividends, properties*	57	90.5
*Family aids (sons)*	1	1.7
*Social assistance*	2	3.3
Social security		
*None*	48	80.0
*Insurance company*	10	16.7
*Free treatment*	2	3.3
Variables	Mean ± SD	(min-max)
Age of caregivers	39.5 ± 9.8	(21–71)
Number of children per house	5 ± 2.0	[1–12]

*USD* United States Dollar, ^a^Income levels are converted from Saudi Riyal (SAR), the local currency, using the conversion rate of $1 = 3.75 SAR

### Characteristics of the caregiving

Analysis of the caregiving patterns showed that 49 (77.8%) participants were constantly available for the patient as primary caregivers and 45 (71.4%) declared being the exclusive caregivers, while 7 (11.1%) declared that their partners were the primary caregivers. The family’s house was the place of care in 52 (82.5%) cases; and 39 (63.9%) reported supporting their children with education and games. The median cost of care expenses was estimated to be 102.5 USD per month per child (range 12.8–384.6 USD). Characteristics of the care giving are presented in Table 2.

**Table 2:** Characteristics of caregiving for children with SCD

Variables	Frequency	Percentage (%)
Primary caregiver		
*Participant*	49	77.8
*Partner*	7	11.1
*Other family member (grandmother, sister)*	2	3.2
*Alternating care (participant & partner)*	5	7.9
Place of caregiving		
*Always home*	52	82.5
*Home and outside*	9	14.3
*Outside*	2	3.2
Caregiver’s assessment of the level of care		
*Satisfactory*	36	57.2
*Acceptable to good*	15	23.8
*Moderately satisfactory*	8	12.7
*Unsatisfactory*	4	6.3
Who is the caregiver?		
*Participant (exclusive)*	45	71.4
*Participant & partner*	4	6.3
*Partner (exclusive)*	11	17.5
*Other family member (grandmother, sister)*	2	3.2
*Nurse*	1	1.6
Educational support		
*Participant*	39	63.9
*Participant & partner*	5	8.2
*Partner*	11	18.0
*Other family member (grandmother, sister)*	4	6.6
*Nurse*	2	3.3
Communication with hospital and school		
*Participant*	35	59.3
*Participant & partner*	5	8.5
*Partner*	17	28.8
*Other family member (grandmother, sister)*	2	3.4
Variable	Median	Quartile
Estimated cost of caregiving (SAR)	400	(50–1500)

### Health assessment of caregivers and their partners

Investigations of medical history showed that 15 (26.2%) caregivers had chronic diseases (14 cases of diabetes, 10 cases of hypertension and 2 cases of SCD). Thirty-five (58.3%) caregivers took medication regularly. As to partners, 10 (16.1%) had chronic disease and 31 (58.5%) took regular medications, according to participants. The median frequency of hospital visits during the last 6 months was 1 visit (range 1–2) in participants and 2 visits (range 1–3) in partners, for various complaints.

### Support received and needed by the caregivers

Most supportive persons were partners, 46 (73.0%) of the cases, followed by children, 24 (38.1%), and grandparents, 16 (25.4%) of the cases. Forty one (65.1%) participants reported having undergone a difficult trial during their life; and 22 (34.9%) among these stated that they needed support to overcome it; and only 13 (20.6%) received a financial support. The data of support received and needed by caregivers are presented in Table 3.

**Table 3:** Support received and needed by caregivers

Variables	Mean ± SD	(min-max)
Level of psychological support received^a^		
*From partner (husband or wife)*	1.9 ± 1.2	(0–3)
*From other relative*	1.6 ± 1.3	(0–3)
*From friend*	0.9 ± 1.3	(0–3)
*From neighbor*	0.8 ± 1.3	(0–3)
*From family doctor*	0.3 ± 0.8	(0–3)
*From social service*	0.1 ± 0.4	(0–3)
	Frequency	Percentage (%)
Most supportive persons		
*Partner (husband/wife)*	46	73.0
*Children*	24	38.1
*Grandparents*	16	25.4
*Sibling*	17	27.0
*Other family member*	7	11.1
*Friend*	11	17.5
Difficult experience	41	65.1
Support needed	22	34.9
Financial support received	13	20.6
*Friend*	9	69.2
*Charity*	2	15.4
*Other*	2	15.4

^a^Level of support calculated on a subjective scale: 0 = absence of support, 1 = weak, 2 = moderate and 3 = strong support

### Daily activities and free time management

Assessment of daily activities and leisure time showed that 24 (38.1%) of the caregivers reported practicing sport activity, especially walking, with a median 1.5 h per week (range 0.5–4.2). The other respondents reported using their free time to watch television (33.3%), read the Quran (14.8%), or go to the internet (18.5%). The mean time spent at home for the caregivers was 20.4 ± 3.3 h per day and 21 (34.4%) were concerned about not finding enough time for themselves; although, 27 (42.9%) reported going to restaurant or coffee and 25 (39.7%) to picnic, on their own time.

### Caregivers’ lifestyle satisfaction

**Fig. 1:**
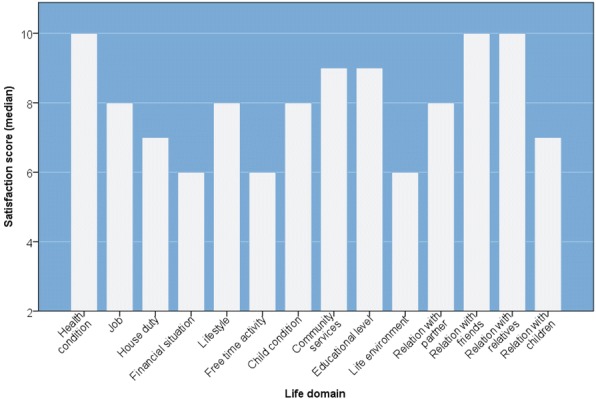
Life style satisfaction among caregivers of children with sickle-cell disease. Subjective assessment of participants’ satisfaction regarding various aspects of their lives: satisfaction levels ranges from 0 (not satisfied at all) to 10 (completely satisfied). Bars represent median satisfaction levels

Participants’ satisfaction regarding various domains of their lives was assessed on a 0 to 10 subjective scale, where 0 = not satisfied at all and 10 = completely satisfied. Results are presented as median scores and are shown in Fig. 1. Reliability testing of this part of the questionnaire on 46 valid observations (excluding the items “job satisfaction” and “education satisfaction” because of their very low response rates) found a Cronbach’s Alpha = 0.762. The most remarkable observations were financial situation, free time activity and life environment being allocated the lowest satisfaction scores (median = 6 each); while the highest satisfaction scores were observed for relationships with friends and relatives and health condition (median score = 10, each).

### Caregivers’ quality of life assessment

Quality of life assessments using the TAAQOL questionnaire showed a range of mean scores, depending on the given dimension. Reliability testing of this part of the questionnaire on 34 valid observations (excluding the item “fine motor skills” because of the very low response rate) found a Cronbach’s Alpha = 0.688. Results showed that emotions, sleep quality and sexual life were the most affected dimensions in caregivers of children with SCD; with median (75th centile) = 44.44 (66.67) for negative emotions, 61.11 (72.22) for positive emotions, 66.67 (91.67) for sleep quality and 50.00 (83.33) for sexual life. Assessments of various dimensions of quality of life of caregivers of children with sickle cell disease are presented in Table 4.

**Table 4:** Assessment of various dimensions of quality of life of caregivers of children with sickle cell disease

S. No	Quality of life dimension	*N*			Quality of life score^a^	
			Range		Mean	SD
1.	Gross motor aptitudes	62	0	100	75.13	27.14
2.	Fine motor aptitudes	10	50.00	100	88.33	17.66
3.	Cognitive skills	62	25.00	100	79.30	22.38
4.	Sleep quality	62	0	100	59.54	32.98
5.	Pain	61	0	100	72.81	29.02
6.	Social contact	61	0	100	73.36	33.36
7.	Professional achievement	49	0	100	81.12	25.95
8.	Sexual life	49	0	100	60.20	24.96
9.	Positive emotions	59	0	100	59.51	20.03
10.	Negative emotions	59	7.41	100	48.15	22.55

^a^The higher the score the better the QoL in the given subscale; *SD* Standard deviation

### Emotional assessments

**Fig. 2:**
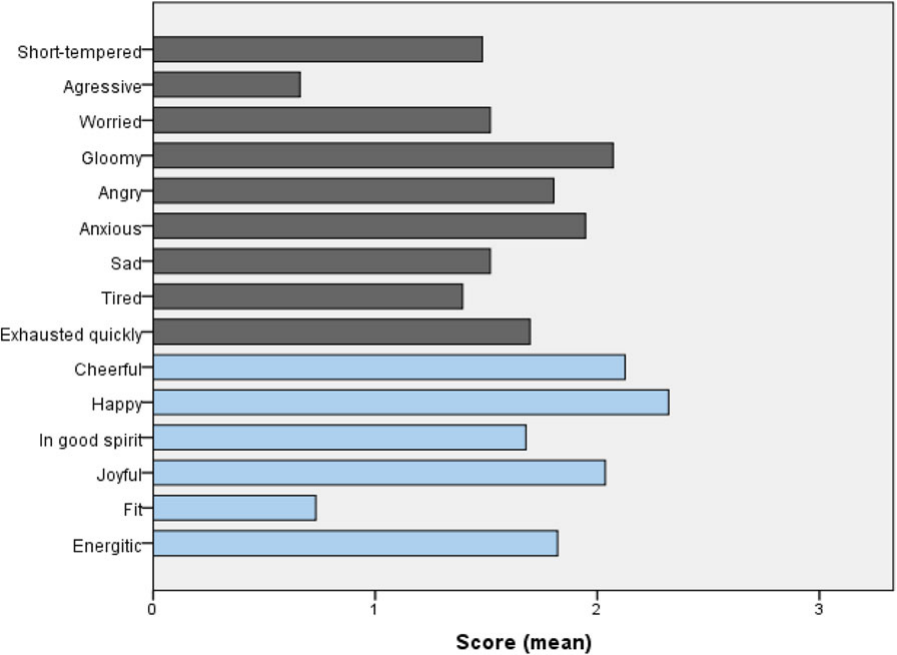
Past month emotional assessment of caregivers of children with sickle-cell disease. Bars represent the mean score of the given feeling, where high scores indicate high frequency/extent of the feeling during the last month (0 = no; 1 = a little; 2 = quite; and 3 = very). Analysis of positive emotions showed high scores of happiness, cheerfulness, and joyfulness; and more than average scores of energetic feeling. Analysis of negative emotions showed relatively high mean scores of gloominess, anxiousness, exhaustion, followed by worry and sadness

Analysis of positive and negative emotions reported during the last month is detailed in Fig. 2. In positive feelings, we observed relatively high extent of happiness, cheerfulness and joyfulness; while in negative ones we observed relatively high extent of anxiousness, gloominess and exhaustion. Reliability testing of this part of the TAQOOL questionnaire on 56 valid observations found a Cronbach’s Alpha = 0.520.

### Risk factors and predictors for impaired quality of life

Age of caregiver showed to be a predictor for gross motor aptitudes, where older age was correlated with worse gross motor aptitudes (B = − 0.94, *p* = 0.006; Additional file 1: Figure S1); however, age was correlated with any of the other QoL dimensions. Mothers, as caregivers, have worse scores regarding negative emotions (median = 40.74) than fathers (64.91) or siblings (68.52), *p* = 0.002 (Kruskal-Wallis test), which was confirmed to be a predictor of bad negative emotion scores in linear regression (B = 16.06, *p* = 0.001); however, no significant difference was observed between the three categories of caregivers in the other QoL dimensions. Surprisingly, number of children showed to be a positive factor for both sleep quality (B = 4.05; *p* = 0.020; Additional file 2: Figure S2) and positive emotions (B = 2.36; *p* = 0.029; Additional file 3: Figure S3). No remarkable differences in the various QoL dimensions were otherwise found across marital status (married versus single or widowed), educational level (illiterate versus up to high school versus university or higher) or monthly income.

### Lifestyle satisfaction as predictor for emotions

Positive and negative emotions were predicted by various aspects of lifestyle satisfaction using univariate linear regression and stepwise regression for multivariate modeling of predictors that are significant in univariate analysis. In univariate models, positive emotions were predicted by the levels of satisfaction of the caregiver of his/her health, job achievement, living conditions and the condition of the diseased child; while negative emotions were mostly predicted by satisfaction of social relations. Life satisfaction scores as predictors for negative and positive emotions are presented in Table 5. In multivariate model, only satisfaction regarding relations with partner and children remained significant predictors for negative emotions (B = 3.86; *p* = 0.003 and B = − 3.65; *p* = 0.032, respectively); while for positive emotions, only satisfaction regarding own health remained a significant predictor (B = 3.89; *p* = 0.025).

**Table 5:** Life satisfaction scores as predictors for negative and positive emotions

S. No	Life domain (predictors)	Positive emotions		Negative emotions	
		B	*p*-value	B	*p*-value
1.	Health condition	2.56	.001^a^	2.48	.050^a^
2.	Job (if applicable)	4.54	.001^a^	2.56	.148
3.	House duty (if housewife)	2.60	.034^a^	− 0.19	.882
4.	Financial situation	1.75	.075	1.22	.274
5.	Life style	2.54	.025^a^	2.07	.110
6.	Free time activity	1.10	.250	0.75	.480
7.	Child condition and quality of care	2.55	.011^a^	1.00	.365
8.	Local services (around home)	−0.06	.947	0.54	.577
9.	Education (if student)	2.39	.045^a^	1.83	.229
10.	Life environment	1.95	.039^a^	1.26	.237
11.	Relation with partner	2.21	.056	3.14	.016^a^
12.	Relation with friends	0.38	.660	2.51	.015^a^
13.	Relation with relatives	0.87	.376	2.69	.016^a^
14.	Relation with children	0.17	.912	−3.10	.079

^a^Statistically significant (*p*-value≤0.05); *B* Unstandardized regression coefficient

## Discussion

The findings of this study highlight the impact of difficulties and challenges faced by close family members of children with SCD, who take care of the patients daily. Family-centered care is part of the continuous, effective and comprehensively coordinated standard health care of patients with SCD [21]. The quality of family-centered care in children with special needs is a critical determinant of healthcare services use [22].

The major observations of this study pointed at the relatively difficult demographic and socioeconomic conditions of caregivers, who were mostly housewife mothers (79.4%) of large families with low financial resources (64.5%) and no social assistance or health insurance (80%). Comparable profiles were described in African caregivers of children with SCD, where 80 to 89.8% were mothers with low occupational status, in households living with less than USD 120 per month in 50.7% of the cases; putting caregivers under huge stress due to inability to cover SCD patient’s needs [12, 17, 19].

Financially, the estimated cost of caregiving (400 SAR = USD 107 in average) was relatively high compared with the socioeconomic conditions of the participants’ families attributing the lowest satisfaction score for financial situation, out of 13 other life domains. Studies from Nigeria, where SCD is highly prevalent, reported an average USD 333 of monthly health expenditure in households with a child afflicted with SCD; which represented up to 34.4% of the family income and significantly impacted finances of 58.2% of the families [9, 23]. Financial stress was described as a significant factor for caregivers difficulty coping with their afflicted children; and the psychosocial impact of the financial burden was more remarkable during SCD crises [2, 23].

On the other hand, most of the tasks and responsibilities related to the caregiving were home-centered and undertaken by the exclusive caregivers. The activities included helping patients to take medications, assisting them during recurrent disease complications and intermittent crises, carrying them to the clinics and hospitals for recurrent blood transfusions or periodic visits, as well as communicating with hospital and school. Consequently, more than half of the interviewees declared lack of time for to take of themselves, exhibiting low participation rates in various daily activities. Similar results were reported in Netherlands, where data indicated a limitation in daily activities of caregivers in relation with the extensive care schedule; including hospital visits, emergency crises and other duties related to SCD child condition [11]. If the importance of the caregiver’s role seems apparent in case of young patients, it was demonstrated to be a significant factor for improving adherence to care even for adolescent patients, who have greater level of autonomy and involvement in their own care [24]. Paradoxically, although being challenging for caregivers, frequent hospital visits for regular blood transfusion therapy are associated with a better QoL of the diseased child. All these factors constitute a permanent pressure on caregivers, which may affect their mental and physical wellbeing, compromising their quality of life and negatively affecting their behaviors and self-respect [19].

Majority of the interviewees (80.9%) declared being fairly or highly satisfied of the level of care provided within the family to their afflicted child. In a society like Saudi Arabia, which is characterized by strong family and spiritual values, home and family-centered care may be a determinant of success in the continuous care of patients with special needs. This is demonstrated by psychological (and financial) support received by caregivers mostly coming from their natural social environment; especially from partners, other family members, friends, and neighbors. Further, life domain satisfaction questionnaire revealed that caregivers are best satisfied of their relations with their children, other relatives’ partners and friends. The relationship between parents and their other, non-diseased, children (siblings of the diseased child) could be dominated by a shift in attention toward the diseased child, a protective attitude regarding the siblings from the pain of their brother/sister, and or their initiation to his/her care; and siblings may develop supportive and responsible attitude in that regard [25].

Assessment of the QoL showed that caregivers of children with SCD have impaired sleep quality and unsatisfactory emotional and sexual life but relatively conserved social and professional achievements. In addition, emotional assessments showed high incidence of anxiousness and feeling of exhaustion and negative trends regarding fitness and energetic feeling; although, a positive trend was observed regarding both happiness and joyfulness feelings. Comparable data were found in two Nigerian studies, where authors reported high proportion of feeling of unhappiness, expressed depressive symptoms, and 2% of suicidal ideations in caregivers. A close monitoring was then recommended with anti-depressive medication [17, 19]. A French study conducted in 2007 evaluated the incidence of post-traumatic stress disorder in 11 children with SCD and their parents following painful crises episodes. The study demonstrated the presence of post-traumatic stress disorders (PTSD) in both afflicted children and their parents. The authors recommended to conduct few more similar studies to address the relation between SCD painful crises and PTSD [15]. In Netherlands, a higher incidence of depressive moods and feeling of unhappiness was found among caregivers which was associated to marked guilt feeling and fear from having another sick child [11]. Furthermore, the same study reported decreased motor and cognitive functioning in caregivers, which was attributed to the lack of sleep resulting from the frequently continuous caregiving, associated sometimes to interrupted sleep pattern, a lack of vigor and vitality [11]. These data are consistent with our findings showing a positive correlation between sleep quality and cognitive skills supposing significant impact of bad sleep quality on cognitive functioning. On the other hand, sleep quality of the caregivers significantly improved in families with more number of children; which was also significantly correlated with more positive emotions among caregivers. These observations further support the effective role siblings can play in taking care of their diseased brother/sister; and suggest that sharing the caregiving with the other family members may be a crucial factor to preserve the QoL of the caregivers, in the long term.

The primary limitation of our study was difficult to reach all patients’ caregivers, due to unavailability of contact details in the patients’ files, in addition to the relatively short timeframe of the study; which resulted in the low participation rate and small sample size. Other aspects limiting generalization of the results include the single-center design, convenience sampling, and non-validation of the Arabic version of the tool. Further, approaching the QoL dimensions with interview-based survey may increase subjectivity in responses and expose to social desirability bias.

## Conclusion

This study was an extension to the previous studies that highlighted the impact of chronic diseases on the families & caregivers of SCD children. The findings of this study demonstrated the huge financial and emotional burdens on the caregivers, who are in need of significant attention and focus in solving their psychosocial difficulties, in order to allow them to give more attention to their sick children and help them to cope up with their illness. The rich social and cultural features of the Saudi society constitute helpful resources for the caregivers of children with SCD, with potentially effective financial and psychological support; however, this should not downplay the role of public institutions and authorities in providing these families with the helping facilities and the required support.

Physicians and healthcare providers should be aware of the huge burdens on caregivers and orient them towards appropriate solutions, to help them meet their needs and improve their QoL and that of their diseased children.

The creation of organizations, groups and networks should be encouraged to support the SCD patients and their families, offering them an environment of free exchange and expression about their daily problems and sufferings and providing them with the useful advice and eventual financial support. Further studies are warranted in Saudi Arabia to investigate the psychosocial burden of SCD on the caregivers.

## Additional files

Additional file 1: **Figure S1.** Linear correlation of gross motor aptitude with age. (DOCX 76 kb)

Additional file 2: **Figure S2.** Linear correlation of sleep quality with the number of children. (DOCX 76 kb)

Additional file 3: **Figure S3.** Linear correlation of positive emotions with the number of children. (DOCX 76 kb)

## Abbreviations

KAUH: King Abdul Aziz University Hospital
PTSD: Post-traumatic stress disorder
QoL: Quality of life
SAR: Saudi Riyals
SCD: Sickle cell disease
TAAQOL: TNO-AZL Questionnaire for Adult’s health-related Quality of Life
