# Supplementary material for: Quality of life among caregivers of sickle cell disease patients: a cross sectional study
Source: Health Qual Life Outcomes. 2018 Sep 10;16:176. doi: 10.1186/s12955-018-1009-5 (PMC6131823; doi:10.1186/s12955-018-1009-5)
Supplement: Supplementary file 1 — Figure S1. Linear correlation of gross motor aptitude with age. (DOCX 76 kb) [file 12955_2018_1009_MOESM1_ESM.docx]

## Additional file 1: Figure S1 Linear correlation of gross motor aptitude with age


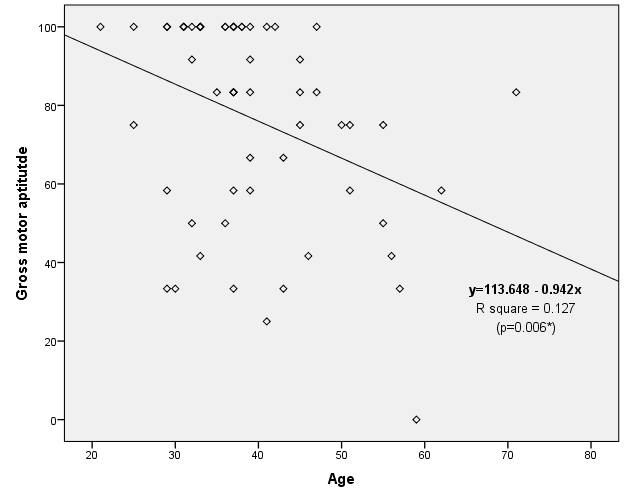


- Age showed to be a predictor for gross motor aptitudes, where older age was correlated with worse gross motor aptitudes (Beta = -0.356, p=0.006)
