# Supplementary material for: Quality of life among caregivers of sickle cell disease patients: a cross sectional study
Source: Health Qual Life Outcomes. 2018 Sep 10;16:176. doi: 10.1186/s12955-018-1009-5 (PMC6131823; doi:10.1186/s12955-018-1009-5)
Supplement: Supplementary file 2 — Figure S2. Linear correlation of sleep quality with the number of children. (DOCX 76 kb) [file 12955_2018_1009_MOESM2_ESM.docx]

## Additional file 2: **Figure S2** Linear correlation of sleep quality with the number of children


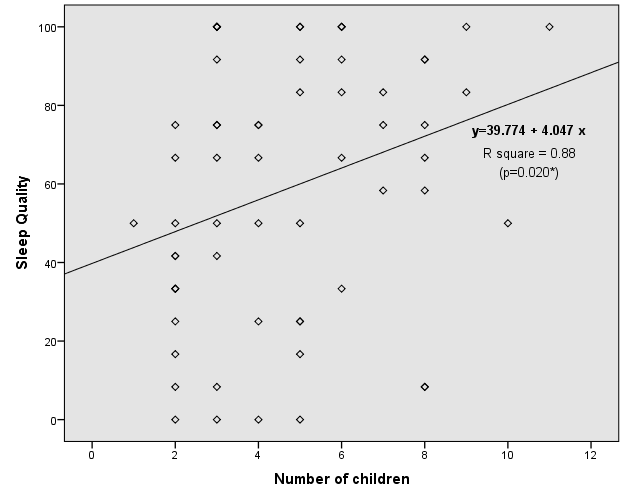


- Surprisingly, number of children showed to be a positive factor for sleep quality (β=0.297; p=0.020).
