# Supplementary material for: Quality of life among caregivers of sickle cell disease patients: a cross sectional study
Source: Health Qual Life Outcomes. 2018 Sep 10;16:176. doi: 10.1186/s12955-018-1009-5 (PMC6131823; doi:10.1186/s12955-018-1009-5)
Supplement: Supplementary file 3 — Figure S3. Linear correlation of positive emotions with the number of children. (DOCX 76 kb) [file 12955_2018_1009_MOESM3_ESM.docx]

## Additional file 3: **Figure S3** Linear correlation of positive emotions with the number of children


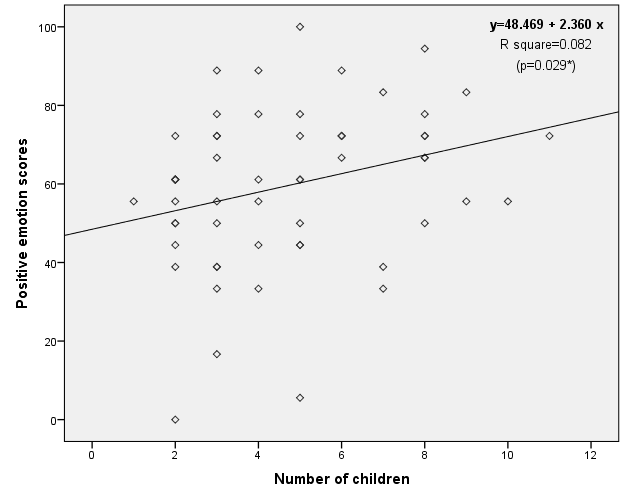


- Number of children showed to be a positive factor for positive emotions (β= 0.286; p=0.029).
